# Using the Program Sustainability Assessment Tool to Assess and Plan for Sustainability

**DOI:** 10.5888/pcd11.130185

**Published:** 2014-01-23

**Authors:** Annaliese Calhoun, Avia Mainor, Sarah Moreland-Russell, Ryan C. Maier, Laura Brossart, Douglas A. Luke

**Affiliations:** Author Affiliations: Avia Mainor, University of North Carolina at Chapel Hill, Chapel Hill, North Carolina; Sarah Moreland-Russell, Ryan C. Maier, Laura Brossart, Douglas A. Luke, Washington University in St Louis, St Louis, Missouri.

## Abstract

Implementing and growing a public health program that benefits society takes considerable time and effort. To ensure that positive outcomes are maintained over time, program managers and stakeholders should plan and implement activities to build sustainability capacity within their programs. We describe a 3-part sustainability planning process that programs can follow to build their sustainability capacity. First, program staff and stakeholders take the Program Sustainability Assessment Tool to measure their program’s sustainability across 8 domains. Next, managers and stakeholders use results from the assessment to inform and prioritize sustainability action planning. Lastly, staff members implement the plan and keep track of progress toward their sustainability goals. Through this process, staff can more holistically address the internal and external challenges and pressures associated with sustaining a program. We include a case example of a chronic disease program that completed the Program Sustainability Assessment Tool and engaged in program sustainability planning.

## Introduction

Public health programs at varied levels and settings struggle with sustaining their efforts over the long term (1–3). Unfortunately, when programs are forced to shut down, hard-won benefits in public health outcomes can dissolve. To maintain these benefits to society, stakeholders need to understand the factors that contribute to program sustainability and look beyond funding (4–6). With knowledge of these critical factors, public health programs can develop a plan to build their capacity for sustainability. We define *sustainability capacity* as the ability to maintain programming and its benefits over time (1). Building this capacity involves developing structures and processes that allow a program to leverage resources to effectively implement evidence-based policies and activities. As a result of increased sustainability capacity in specific organizational and contextual factors, programs can perform more efficiently and improve their ability to maintain efforts over the long term (7). Regardless of a program’s age and experience, sustainability is always important (8). Beginning with crafting a logic model, and extending throughout the project lifecycle, sustainability needs to be on the mind of program staff and evaluators (2,4,9). Through creating and implementing a sustainability plan, programs can be better positioned for long-term success (10,11).

The Center for Public Health Systems Science (CPHSS) at Washington University in St Louis developed the Sustainability Framework and the Program Sustainability Assessment Tool (PSAT) to address the lack of reliable sustainability measurement tools (4,5). Through a literature review, concept mapping, and expert input, CPHSS identified 8 domains affecting a public health program’s sustainability capacity. These 8 domains comprise the Sustainability Framework (Table 1) (1). The PSAT is a reliable tool to assess a program’s sustainability capacity across these 8 domains (12).

**Table 1:** Program Sustainability Framework Domains and Definitions

Domain	Definition
Political Support (now called Environmental Support)	Having a supportive internal and external climate for your program
Funding Stability	Establishing a consistent financial base for your program
Partnerships	Cultivating connections between your program and its stakeholders
Organizational Capacity	Having the internal support and resources needed to effectively manage your program
Program Evaluation	Assessing your program to inform planning and document results
Program Adaptation	Taking actions that adapt your program to ensure its ongoing effectiveness
Communications	Strategic communication with stakeholders and the public about your program
Strategic Planning	Using processes that guide your program’s directions, goals, and strategies

This article provides a step-by-step guide (Figure) for how a program can use the PSAT to identify its strengths and challenges and engage in sustainability planning to build sustainability capacity. We also provide a case example of the use of the PSAT.

**Figure:**
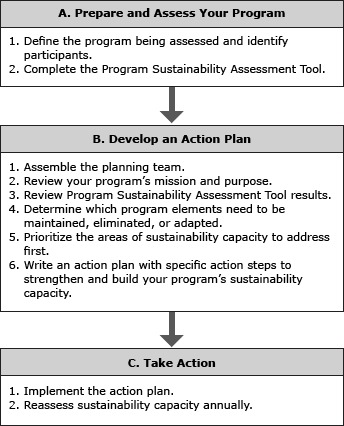
The program sustainability planning process.

## Using the Program Sustainability Assessment Tool and Engaging in Sustainability Action Planning

### A. Prepare and assess your program

#### 1. Define the program being assessed and identify participants

The first step in assessing program sustainability capacity entails defining the program you wish to assess. The program is the main unit of analysis; thus, it is important that all participants have a clear understanding of the program they are assessing. This is especially important if external partners or stakeholders are invited to participate or if you are using the PSAT to assess a set of loosely organized activities or a coalition rather than a named program with prescribed activities. Defining what is meant by program ensures that everyone assesses the same set of activities (13,14). The PSAT also includes questions about the organization where the program is housed, so it is important to clearly define the organization (eg, the name of the health department or nonprofit agency).

In the first step, you should identify the people who will complete the PSAT. Engaging the appropriate staff and stakeholders and ensuring you have organizational and programmatic leadership buy-in are essential for the success of the sustainability planning process (6,8). We recommend that multiple staff from a program complete the PSAT (the number depends on program setting and size); if community support or partners are essential for the services provided, consider involving representatives from these groups. When identifying participants for the PSAT, choose people who are knowledgeable about the program and invested in its mission. For example, frontline staff, volunteers, program managers, organizational leadership, and budget officers could all bring different, but valuable, perspectives. Including responses from a wide array of people ensures a fuller perspective of the strengths and challenges of a program’s sustainability capacity. We also recommend that each participant complete the assessment separately and anonymously, to encourage honesty among staff and stakeholders at all levels.

#### 2. Complete the Program Sustainability Assessment Tool

Once the program and stakeholders are defined, you are ready to invite participants to complete the assessment. You can complete the PSAT in paper form or online at https://sustaintool.org. There are 40 questions total (5 questions in each of the 8 domains) (Table 1). For each question, participants are asked to rate the program on the presence of a particular element by using a scale of 1 (to little or no extent) to 7 (to a great extent).

If you are using the sustaintool.org website, the site will automatically calculate the scores for each individual and for the group as a whole via an automated summary Sustainability Report. If you are analyzing the group results yourself, use the following steps:

1. Question Scores: For each question for which you received responses, calculate the average score across all group members. For example, if you have 7 group members, calculate the average of 7 responses for each question. (Assuming that all questions were answered, you should have 40). Do not include “not able to answer” responses in calculating your averages.
2. Domain Scores: Group your 40 question scores by domain. (You should have 5 scores in each domain.) Calculate the average across these 5 scores for each of the 8 domains.
3. Calculate the average of your 8 domain scores.
4. Share your scores in a Sustainability Report (see a sample Sustainability Report at https://sustaintool.org/assess).

### B. Develop an Action Plan

#### 1. Assemble the planning team

As with any planning process, you need to have the right people in the room and a clear agenda for guiding discussion. Not everyone who takes the PSAT needs to be involved in the planning process. However, it would be helpful if those involved have taken the PSAT so that their opinions are represented in the sustainability scores. Consider selecting people who are in leadership roles or who are key to the program’s operations and who can commit to the process of sustainability planning. The number will vary depending on the program’s setting and structure.

#### 2. Review your program’s mission and purpose

Once the planning team is assembled, take time to revisit the program’s mission or long-term objectives. You can refer to your program logic model or strategic plan for the short-, medium-, and long-term outcomes you are trying to achieve. Specify what a sustainable program would look like 3 or 5 years from now and identify program goals. Having a clear vision of your program’s purpose will help you unite program staff, organizational leadership, and other stakeholders. This step will also provide a greater understanding of the effort needed to maintain the program and its benefits over time (8,14,15).

#### 3. Review Program Sustainability Assessment Tool results

Next, as a team, review the PSAT summary Sustainability Report. This report provides a snapshot of the program’s current sustainability capacity. Discuss surprises in the results and differences in opinion about ratings. Remember that there is no score that can guarantee a program’s sustainability. However, low scores in certain domains suggest areas that need attention. Domain scores are meant to serve as starting points for discussion rather than endpoints.

#### 4. Determine which program elements need to be maintained, eliminated, or adapted

Considering your program goals and PSAT scores, determine which elements of your program should be sustained and which could or should be dropped. Do not assume that all program components should be continued exactly as is into the foreseeable future (6). This is the time to compare your program’s ideal future (identified in Step B2) with the reality of its current level of sustainability (identified by PSAT in Step B3) and find ways to bridge the difference (2). As the Program Adaptation domain highlights, programs must be able to respond to changes in the environment as well as population needs. Research suggests that, regardless of a program’s ability to support continued implementation of an activity, it is unlikely to be sustained if it does not meet the needs of the intended audience (4). Consider some of the following questions and draw on evaluation data as you decide what should be sustained (6):

- Is your program providing crucial services to the community?
- Is your program effectively meeting the needs of its intended audience?
- Can your program demonstrate public health impact or positive effects on its target outcomes?
- Are key stakeholders committed to continuing your program?
- If your program were to suffer a drastic funding cut in the future, do you know which elements you would cut and which core services you would continue?

At this point in the process, it is important to discuss which pieces of your program should or should not continue in order to best support your program’s long-term sustainability efforts.

#### 5. Prioritize the areas of sustainability capacity to address first

Once you have clarified the future direction and scope of your program, it is time to prioritize which sustainability domains and indicators your team will address first. Review areas of weakness within your sustainability results and decide which ones are most urgent (refer to your discussion from steps B3 and B4). Consider where you might achieve the biggest payoff with the least amount of resources. Discuss which sustainability indicators are most modifiable for your program. Given your time and resources, prioritize the domain(s) or specific indicator(s) your team wants to address. Depending on your program’s context, certain domains and items may be more important than others. If it is very difficult to identify your program’s long-term goals or locate sufficient evaluation data to decide which program elements to drop, it may be wise for your team to begin by addressing the Strategic Planning and Program Evaluation domains.

#### 6. Write an Action Plan

When developing an Action Plan that is based on the prioritized list, your team can make the plan as simple or as detailed as necessary. This sustainability plan will serve as a roadmap for building your program’s sustainability capacity and can provide institutional memory about why and how actions were determined and achieved. At a minimum, we suggest that your written plan for building sustainability capacity include the following items:

**a) Action steps for each priority domain. **Action steps should clearly describe how you will address the priority sustainability areas you identified earlier (see Step B5). Be realistic in your action steps, keeping in mind the current political and organizational environment in which you are working (15,16). Each action step should include a person responsible for overseeing the step, along with a timeframe for completion.

**b) List of resources and stakeholders required to make action steps successful. **To execute your sustainability plan, you may need specific resources, including time, money, space, materials, technology support, and in-kind resources. In addition, there may be specific staff, stakeholders, or partners who need to be involved in completing an action step. As you write the plan, identify the resources, people, or groups that are needed to accomplish outlined action steps. Consider the unique skills and resources that different partners and stakeholders contribute to your program and how they might help advance your sustainability efforts. Studies indicate that it is vital to have influential and proactive leaders and champions, both inside and outside of your program (4,17–19). These supportive advocates are essential for creating a setting that promotes and facilitates sustainability (4,8,17,18).

**c) Methods for tracking progress toward the completion of your action steps. **Your sustainability plan should identify how you will monitor and track progress on each action step. Consider the metrics you will use to measure success. Having a tracking process determined in advance ensures that you document your progress appropriately.

As you build your plan, try to foresee any opposition you may face along the way, internally and externally (16). Discuss possible steps to take to win over opponents, navigate the system, or reduce the effects of opposing forces.

### C. Take action

#### 1. Implement the Action Plan

Developing a sustainability plan is not a one-time effort. Your team must continue working together to implement the plan over the weeks and months following the assessment. Use the plan to guide the ongoing management of program activities. Discuss the action steps and activities with program staff and stakeholders by providing periodic updates and collectively brainstorming solutions to challenges encountered along the way (6,8). Monitor your progress toward completing each action step.

#### 2. Reassess sustainability capacity annually

Building your program’s sustainability capacity will take time. Assessing your program’s sustainability capacity, identifying strengths and challenges, and taking concrete steps to improve your sustainability capacity are all essential steps in this process. Consider completing the PSAT annually as part of yearly progress reporting and compare the scores to monitor improvements (11). Celebrate gains in your sustainability capacity and adjust your sustainability plan to address areas of new or continued weakness (8).

## Case Example: Program Creates Communications Sustainability Action Plan

In the spring of 2011, a state health department faced a number of rapid changes, including the possibility of integrating numerous chronic disease programs within the unit. With continued funding uncertain, one of the chronic disease program directors decided to organize a team of staff and unit managers to complete the PSAT and assess their program’s sustainability capacity.

Team members completed the PSAT as a group. After reviewing the program’s Sustainability Report, the group quickly recognized the need to involve stakeholders in this process. The group reached out to the health department’s upper management to ensure that they understood the department’s priorities and how these might impact the chronic disease program. Key community partners, coalition members, and other health department program managers were also invited to take part in the sustainability planning process.

The team committed a considerable amount of time to the planning. First, the team completed a visioning process that included a review of the program’s mission and overall purpose with a focus on the desired outcomes. This process helped determine which program components were necessary to maintain the program and sustain its benefits over time. Next, the team reviewed their Sustainability Report and agreed to address the Communications domain first. Program staff believed they had done a poor job of demonstrating their accomplishments over the last 3 years, which was reflected in low scores on the following Communications indicators:

- The program has communication strategies to secure and maintain public support.
- Program staff communicates the need for the program to the public.
- The program demonstrates its value to the public.

The scores indicated that the program’s communications had been haphazard and reactionary instead of strategically planned; communications efforts also did not make use of current technology. The team saw that increasing communication capacity would be critical for documenting their success and reach to funders (currently and in the future), engaging stakeholders, and ultimately promoting their long-term sustainability.

Through the planning process, the team realized that the lack of a dedicated communications manager limited their ability to market and publicize the program’s vision, generate public support, and engage new partners. Because the program did not have funds to hire a dedicated communications manager, the team developed 4 communications-focused action steps to improve their sustainability capacity in this area (Table 2). For each of the 4 steps, they designated who would be responsible, determined the resources needed to accomplish the steps, and developed a monitoring plan. The long-term goal of their plan was to identify strategies to communicate the impact and value of their program to the community.

**Table 2:** Sample Sustainability Action Plan Addressing the Communications Domain

Action Steps (Time Frame)	Person or Group Responsible	Resources	Monitoring Progress
1. Establish a communications team within the unit that will meet monthly to develop an integrated communications plan including goals, objectives, and strategies for implementation. (Communications team established by January 1st. Meet on the second Wednesday of each month)	• Unit directors — must appoint staff person to serve as representative • A chairperson will be selected by team members	• Representatives from chronic disease programs (work plan requirement) • Monthly meeting time • Communications training workshop	• Established communications team • Documented timelines and milestones in meeting notes • Completed communications plan
2. Create quarterly information graphics to demonstrate the program’s impact to senior leadership and external partners. (March 201x–December 201x)	Communications team chairperson and program manager	• Graphics software • Partnership meeting summaries and evaluation data • Staff time to present to leadership	• Data queries • Distribution of quarterly information graphics
3. Improve storytelling capacity through better organization, collection, and training of health department staff, local partners, and grantees. Training will include face-to-face meetings, webinars, and resources. (Beginning May 201x–ongoing)	Health department public information staff	• Storytelling train-the-trainer workshops • Travel costs, staff time, printing costs • Ongoing technical assistance to partners and grantees • Tracking database	• Training curriculum • Attendance list from trainings • Report of changes or implemented strategies following training events
4. Develop 5 short multimedia success story vignettes communicating the value of the program for use in community presentations, on the website, and social media outlets. (Completed by September 201x)	Website and Facebook administrator	• Editing and graphics support • Success story mining from grantee reports and evaluation data • Implementation pictures from site visits	• Creation of success story vignettes • Distribution of stories • Delivery of presentations, web posts, blog announcements, tweets, etc.

The team developed a database to track the progress of their action steps. The team decided to monitor and reassess their sustainability capacity during their annual reporting cycle to ensure continued improvement of their sustainability capacity.

The chronic disease program staff benefited from the sustainability planning process in a number of ways. First, it provided an overview of their current sustainability strengths and weaknesses. Second, it helped them determine which program elements they intended to sustain for the long term. Third, it facilitated the development of a vision and mission for the program to reference and use as they monitored the progress of their actions. Lastly, the Communications Action Plan enabled them to demonstrate their accomplishments, tell their story, and build connections to support their sustainability beyond dollars.

## Limitations and Implications for Practice

Many factors affect a public health program’s capacity for sustainability. Although funding is important, other factors such as strong organizational support, partnerships, and communications can help make a program more competitive for funding, more successful in program work, and better able to weather challenges.

The PSAT and planning process provide public health programs and their partners with a reliable method for rating their programs’ capacity for sustainability. This method is unique in that programs are able to identify their areas of strength and weakness across a range of sustainability factors and then make informed decisions about where to concentrate efforts.

One advantage of the PSAT is that it is a simple, user-friendly tool that can be used in multiple settings and fields; already, more than 550 public health practitioners and managers at the community and state levels have used the PSAT to assess their programs (12). However, the brevity and simplicity of the PSAT are also limitations, because there will be nuances of setting and situation that the PSAT cannot capture. In these cases, it will be important to have discussions as a team about the domains as a whole, rather than limiting discussions to the 5 indicators of sustainability capacity identified for each domain in the PSAT.

A slightly modified version of the PSAT was rolled out in late 2013. In this PSAT V2, items within the Political Support domain were adapted and the domain name was changed to Environmental Support (Table 1). The updated domain will appear at https://sustaintool.org, along with more information about this transition.

We hope that those who follow these steps will gain a greater understanding of how to engage their program teams in an assessment of their program’s capacity for sustainability and in sustainability action planning. To ensure the long-term success and impact of public health programs, managers and practitioners must engage in ongoing sustainability assessment and planning.
